# Evidence to Support Karyotypic Variation of the Mosquito, *Anopheles peditaeniatus* in Thailand

**DOI:** 10.1673/031.011.0110

**Published:** 2011-02-03

**Authors:** Wej Choochote

**Affiliations:** Department of Parasitology, Faculty of Medicine, Chiang Mai University, Chiang Mai 50200, Thailand

**Keywords:** COI, COII, crossing experiment, Hyrcanus group, ITS2, metaphase karyotype

## Abstract

Eight isoline colonies of *Anopheles peditaeniatus* Leicester (Diptera: Culicidae) were established from wild-caught females collected from buffalo-baited traps at 8 localities in Thailand. They showed 2 types of X (X_2_, X_3_) and 4 types of Y (Y_2_, Y_3_, Y_4_, Y_5_) chromosomes based on the number and amount of major block(s) of heterochromatin present in the heterochromatic arm, and were tentatively designated as Forms B (X_2_, X_3_, Y_2_), C (X_3_, Y_3_), D (X_3_, Y_4_) and E (X_2_, X_3_, Y_5_). Form B was found in Nan, Ratchaburi, and Chumphon provinces; Form C was obtained in Chon Buri province; Form D was recovered in Kamphaeng Phet province; and Form E was acquired in Chiang Mai, Udon Thani, and Ubon Ratchathani provinces. Crossing studies among the 8 isoline colonies, which were representative of 4 karyotypic forms of *An. peditaeniatus*, revealed genetic compatibility in providing viable progenies and synaptic salivary gland polytene chromosomes through F_2_-generations, thus suggesting the conspecific nature of these karyotypic forms. These results were supported by the very low intraspecific sequence variations (0.0 – 1.1%) of the nucleotide sequences in ribosomal DNA (ITS2) and mitochondrial DNA (COI and COII) of the 4 forms.

## Introduction

The Hyrcanus group of the Myzorhynchus series of the subgenus *Anopheles* (Diptera: Culicidae) comprises a large number of species that occur widely in Asia. At least 8 species of this group, i.e. *Anopheles argyropus* Swellengrebel, *An. crawfordi* Reid, *An. nigerrimus* Gilles, *An. nitidus* Harrison, Scanlon and Reid, *An. paraliae* Sandosham, *An. peditaeniatus* Leicester, *An. pursati* Laveran, and *An. sinensis* Wiedemann are recorded in Thailand (Harrison and Scanlon 1975; Rattanarithikul et al. 2006). Among these, *An. nigerrimus, An. peditaeniatus*, and *An. sinensis* are suspected as vectors of *Plasmodium vivax* Grassi and Feletti in Thailand (Harrison and Scanlon 1975; Rattanarithikul et al. 1996), while *An. sinensis* has been incriminated as a natural vector of *P.* *vivax* in Korea (Chai 1999; Ree et al. 2001) and *An. peditaeniatus* as a secondary vector of Japanese encephalitis virus in China and India (Mourya et al. 1989; Zhang 1990; Kanojia et al. 2003). Although *An. peditaeniatus* has been found abundantly and widely distributed throughout Thailand, its status as a vector of the Japanese encephalitis virus remains a crucial question that needs to be clarified more thoroughly. Additionally, this species was also considered an economic pest of cattle because of its vicious biting-behavior and ability to transmit cervid filariae of the genus *Setaria* (Reid 1968; Harrison and Scanlon 1975).

Regarding the cytogenetic investigations of *An. peditaeniatus*, the results indicated that at least 3 types of X (X_1_, X_2_, X_3_) and 5 types of Y (Y_1_, Y_2_, Y_3_, Y_4_, Y_5_) chromosomes were found in both sympatric and/or allopatric populations in Chanthaburi, Chiang Mai and Phrae provinces (Baimai et al. 1993).

Chromosomes X_1_, X_2_ and X_3_ differ from each other in the number and amount of major block(s) of heterochromatin present in the heterochromatic arm, making them appear as metacentric X_1_, small submetacentric X_2_, and large submetacentric X_3_ chromosomes. Likewise, the evolution of Y chromosome types, i.e. very small telocentric Y_1_, medium telocentric Y_2_, large telocentric Y_3_, very large telocentric Y_2_, and submetacentric Y_5_ could have arisen via the process of gain, rather than loss, of major block(s) of heterochromatin (Baimai et al. 1993; Baimai 1998). Although marked genetic variation at the chromosomal level of *An. peditaeniatus* has obviously been illustrated, little is known about its genetic proximities. Accordingly, the chromosomal variant and/or distinction might be manifested as an important role in generating post-mating barrier and DNA sequence variation of some specific genomic regions. Thus, this paper presents the results of crossing experiments and comparative DNA sequencing of the ribosomal DNA (ITS2) and mitochondrial DNA (COI and COII) regions of 4 karyotypic forms of *An. peditaeniatus* strains from 8 localities in Thailand.

## Materials and Methods

### Field collections and the establishment of isoline colonies

**Figure 1.**
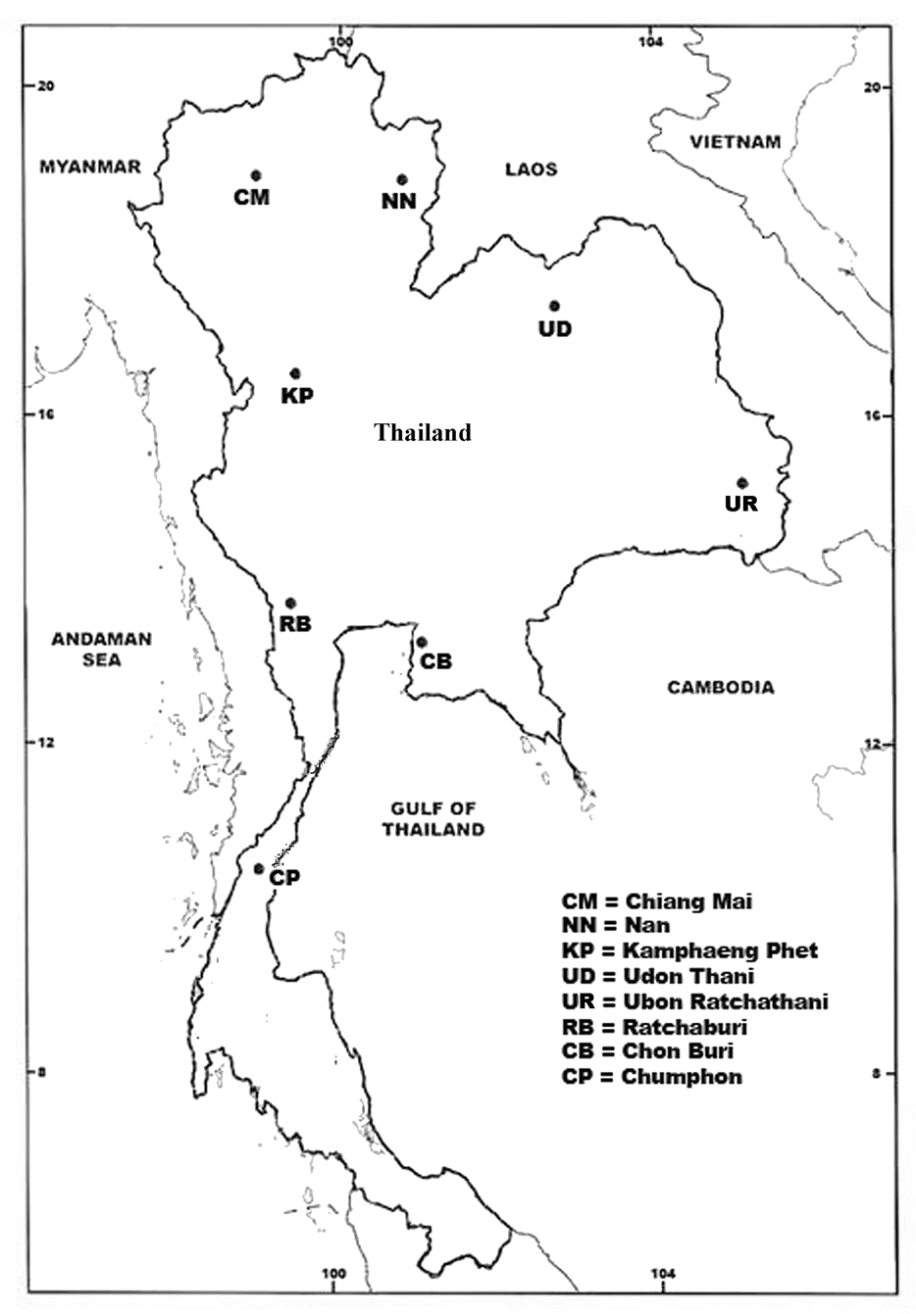
Map of Thailand showing 8 locations where mosquitoes were collected; northern region: Chiang Mai and Nan provinces; northeastern region: Udon Thani and Ubon Ratchathani provinces; central region: Kamphaeng Phet province; western region: Ratchaburi province; eastern region: Chonburi province; and southern region: Chumporn province. High quality figures are available online.

Wild, fully engorged female *An. peditaeniatus* were collected from buffalo-baited traps from November 2007 to September 2008 at 8 localities in Thailand (Figure 1; Table 1). Eight isoline colonies were successfully established and maintained in an insectary using the techniques described by Kim et al. (2003). These isoline colonies were used for studies on metaphase karyotypes, crossing experiments, and molecular analyses.

### Metaphase chromosome preparation

Metaphase chromosome preparation was the technique used for chromosome preparation in adult mosquitoes, as described by Choochote et al. (2001). Briefly, newly emerged adult males of laboratory-raised *An. peditaeniatus* (aged about 6–12 hr) were intra-thoracically inoculated with 0.30 µl of 1% ethanol-extracted *Gloriosa superba* L. (Liliales: Colchicaceae) solution and held in an insectary at 27 ± 2°C, with 70–80% relative humidity for 3 hr. The excised testes were incubated in 1% hypotonic sodium citrate solution, fixed in Carnoy's fixative, stained with 10% Giemsa in phosphate buffer pH 7.2, mounted in Permount® (Fisher, www.fishersci.com), and examined under a compound microscope. Identification of types of sex chromosomes followed the cytotaxonomic key of Baimai et al. (1993).

**Table 1.**
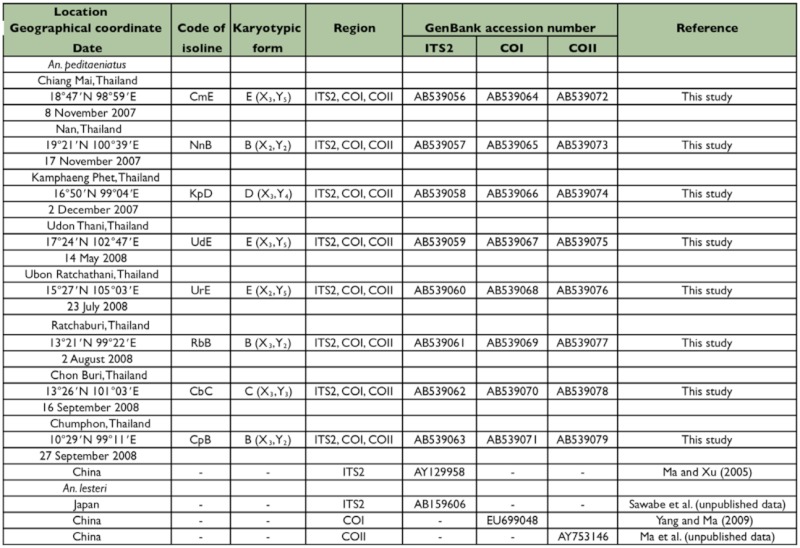
Locations, isoline colonies and karyotypic forms of Anopheles peditaeniatus and An. lesteri, and their GenBank accession numbers.

### Crossing experiments

In crossing experiments, the 8 laboratoryraised isoline colonies of *An. peditaeniatus* were representative of the 4 karyotypic forms, i.e. Forms B [Nan strain: NnB (X_2_, Y_2_), Ratchaburi strain: RbB (X_3_, Y_2_), Chumphon strain: CpB (X_3_, Y_2_)], C [Chon Buri strain: CbC (X_3_, Y_3_)], D [Kamphaeng Phet: KpD (X_3_, Y_4_)], and E [Chiang Mai strain: CmE (X_3_, Y_5_), Udon Thani strain: UdE (X_3_, Y_5_), Ubon Ratchathani strain: UrE (X_2_, Y_5_)] (Table 1). These isoline colonies were used for crossing experiments in order to determine post-mating reproductive isolation by employing the techniques previously reported by Thongsahuan et al. (2009). Experiments were carried out once for each crossing of karyotypic forms. The salivary gland polytene chromosomes of 4^th^ instar larvae from the crosses were investigated using the techniques described by Kanda (1979).

### DNA extraction, amplification, sequencing and analysis

One individual F_1_-progeny adult female from each isoline colony of *An. peditaeniatus* forms was used for DNA extraction and amplification. Genomic DNA was extracted from individual adult mosquitoes using a RED Extract-N-Amp™ Tissue PCR Kit (Sigma-Aldrich). The ribosomal DNA (rDNA) internal transcribed spacer 2 (ITS2), and mitochondrial cytochrome *c* oxidase subunit I (COI) and subunit II (COII) were amplified using the primers described by Park et al. (2003), with minor modifications: 5.8S + 35 (5′-ACG CAT ATT GCA CGT CGT GG-3′) and 28S - 20 (5′-GGG TTG TCA CAC ATA ACT TGA GGC-3′) for ITS2; LCO1490 (5′GGT CAA CAA ATC ATA AAG ATA TTG G-3′) and HCO2198 (5′-TAA ACT TCA GGG TGA CCA AAA AAT CA-3′) for COI; AnoCO2+1 (5′-GAT TAG TGC AAT GAA TTT AAG C-3′) and AnoCO2END (5′-GAG ATC ATT ACT TGC TTT CAG TC-3′) for COIL The PCR condition, cloning, and sequencing followed the techniques previously reported by Park et al. (2008). The PCR products were purified using the QIAquick® Gel Extraction Kit (Qiagen, www.qiagen.com), and directly sequenced with an ABI PRISMH® 3700 DNA Analyzer (Applied Biosystems, www.appliedbiosystems.com) using a Dye Terminator Cycle Sequencing Ready Reaction Kit (Applied Biosystems). Both strands were sequenced and aligned using the ClustalX multiple alignment programs (Thompson et al. 1997). The nucleotide sequence data reported are in the DDBJ/EMBL/GenBank nucleotide sequence databases with the accession numbers AB539056-AB539079. Geographical type of specimens and their sequence accession numbers within GenBank are denoted in Table 1.

**Figure 2.**
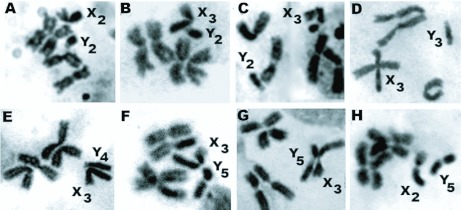
Metaphase karyotypes of Anopheles peditaeniatus. Form B: (A) Nan strain: showing X_2_, Y_2_ chromosomes; (B) Ratchaburi strain: showing X_3_, Y_2_ chromosomes; (C) Chumphon strain: showing X_3_, Y_2_ chromosomes. Form C: (D) Chonburi strain: showing X_3_, Y_3_ chromosomes. Form D: (E) Kamphaeng Phet strain: showing X_3_, Y_4_ chromosomes. Form E: (F) Chiang Mai strain: showing X_3_, Y_5_ chromosomes; (G) Udon Thani strain: showing X_3_, Y_5_ chromosomes; (H) Ubon Rathchathani strain: showing X_2_, Y_5_ chromosomes. High quality figures are available online.

## Results

Cytological observations of F_1_-progenies of 8 isoline colonies demonstrated 2 types of X (X_2_, X_3_) and 4 types of Y (Y_2_, Y_3_, Y_4_, Y_5_) chromosomes. Based on uniquely different characteristics of Y chromosome from each isoline colony, they were tentatively designated as Forms B (X_2_, X_3_, Y_2_), C (X_3_, Y_3_), D (X_3_, Y_4_), and E (X_2_, X_3_, Y_5_). Form B was detected in 3 isoline colonies from Nan (X_2_, Y_2_), Ratchaburi (X_3_, Y_2_), and Chumphon (X_3_, Y_2_) provinces. Form C was found in 1 isoline colony from Chonburi (X_3_, Y_3_) province. Form D was obtained in 1 isoline colony from Kamphaeng Phet (X_3_, Y_4_) province. Form E was recovered in 3 isoline colonies from Chiang Mai (X_3_, Y_5_), Udon Thani (X_3_, Y_5_) and Ubon Ratchathani (X_2_, Y_5_) provinces (Figure 2; Table 1).

**Figure 3.**
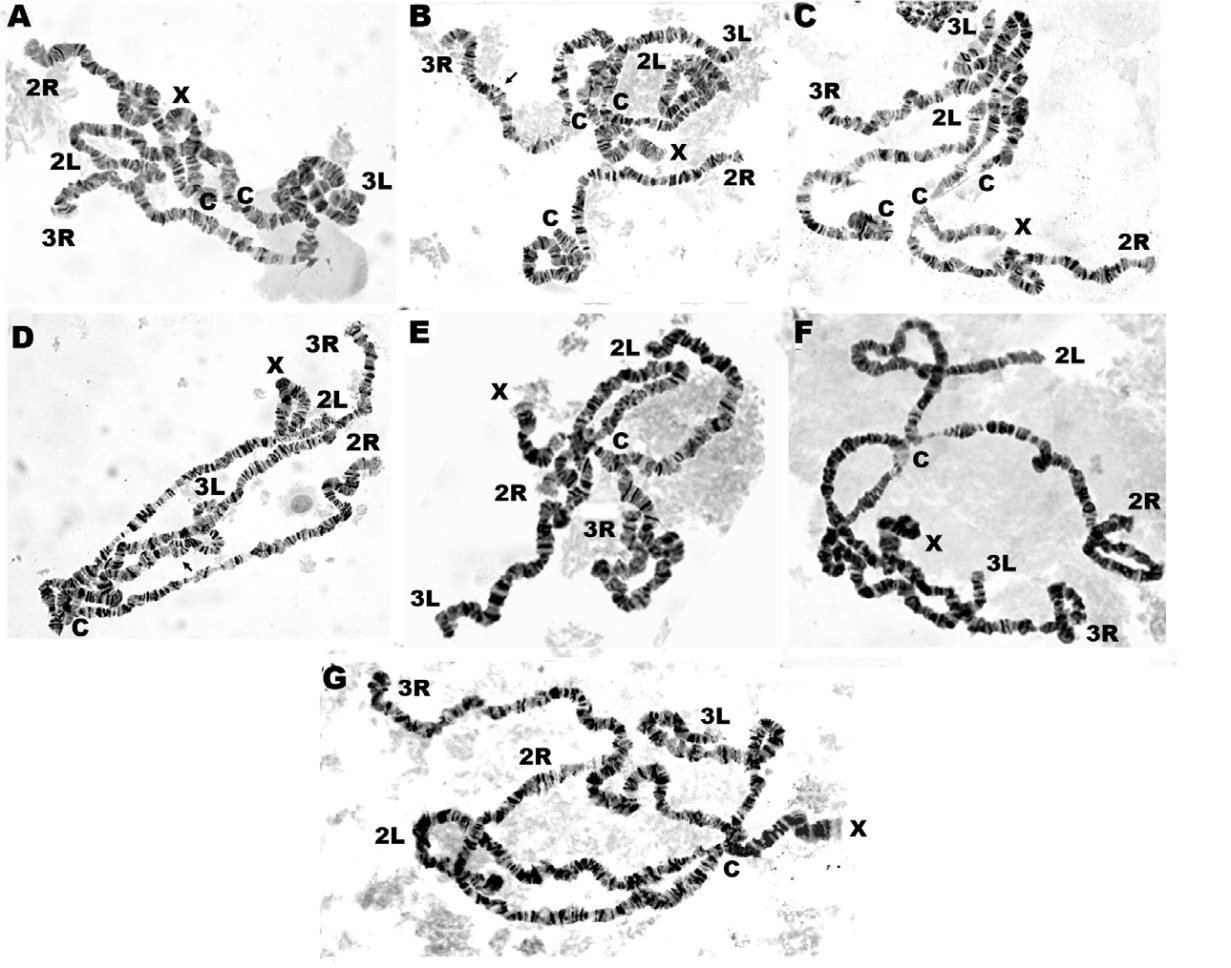
Complete synapsis in all arms of salivary gland polytene chromosome of F_1_-hybrid 4^th^ stage larvae of Anopheles peditaeniatus. (A) NnB female × CpB male; (B) NnB female × RbB male, note: small gap of homosequential asynapsis was found on chromosome 3R; (C) NnB female × CbC male; (D) NnB female × KpD male, note: small gap of homosequential asynapsis was found on chromosome 3L; (E) NnB female × CmE male; (F) NnB female × UdE male; (G) NnB female × UrE male. High quality figures are available online.

For crossing experiments, details of hatchability, pupation, emergence, and adult sex-ratio of parental, reciprocal, and F_1_-hybrid crosses among the 8 isoline colonies of *An. peditaeniatus* Forms B (X_2_, X_3_, Y_2_), C (X_3_, Y_3_), D (X_3_, Y_4_), and E (X_2_, X_3_, Y_5_) are shown in Table 2. All crosses yielded viable progenies through F_2_-generations. No evidence of genetic incompatibility and/or post-mating reproductive isolation was observed among these crosses. The salivary gland polytene chromosomes of the 4^th^ stage larvae from all crosses showed complete synapsis along the whole length of all autosomes and the X chromosome (Figure 3).

**Figure 4.**
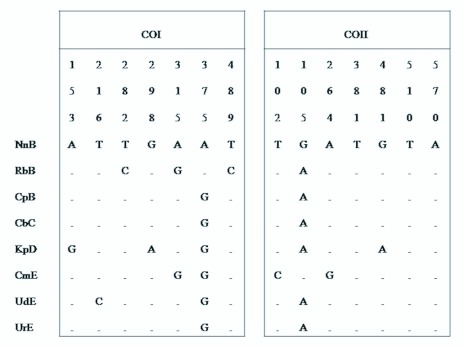
Variable sites in the sequence alignment of the COI and COII sequences. Bases are numbered relative to the alignment. Only those positions differing from the consensus are shown. A dot indicates a base pair identical to that of the NnB sequence. Sequence names are defined in Table 1. High quality figures are available online.

In the DNA sequence analysis, DNA sequences were determined and analyzed for the ITS2, COI, and COII regions from 8 isoline colonies representative of 4 karyotypic forms of *An. Peditaeniatus.* In these, all sequences of the ITS2 region were found to be completely identical with a length of 463 bp, but in comparison with *An. lesteri* they had a very high interspecific sequence variation of 35.4%. The results of comparative sequences of COI and COII regions revealed 548 bp for COI with 0.0 – 1.1% intraspecific sequence variations, and 672 bp for COII with 0.0 – 0.8% intraspecific sequence variations, and seven variable sites were observed from both (Figure 4). Interspecific sequence variations between *An. peditaeniatus* and *An. lesteri* in COI and COII were 3.6 – 4.0% and 3.1 – 3.5%, respectively.

## Discussion

Karyotypic variation, due to the addition of an extra block of herterochromatin on sex chromosome (X, Y), is an important mechanism in the speciation process of anopheline mosquitoes and/or other dipteran insects. It could be used as a primary marker for further investigations of sibling species or subspecies status in natural populations of mosquitoes, particularly in those that have heteromorphic sex chromosomes as anophelines (Baimai 1998; Subbarao 1998). Nonetheless, limitation in use should be kept in mind since either markedly different or identical metaphase karyotypes could be cytological characteristics of sibling species or subspecies (cytological races). For example, *An. minimus* Theobald (*minimus* species A) has uniquely submetacentric X_1_, medium submetacentric X_2_, and submetacentric Y_1_ chromosomes. *Anopheles harrisoni* Harbach and Manguin (*minimus* species C) has unique large submetacentric X_3_ and large submetacentric Y_2_ chromosomes (Baimai et al. 1996). *Anopheles barbirostris* Van der Wulp species A1, A2, A3, and A4 share common characteristics of medium submetacentric X_2_ and subtelocentric Y_1_ chromosomes, whereas submetacentric X_1_, large submetacentric X_3_, submetacentric Y_2_, and large submetacentric Y_3_ chromosomes were common phenomena of the karyotypic variation of *An. barbirostris* species A1 (Suwannamit et al. 2009).

**Table 2.**
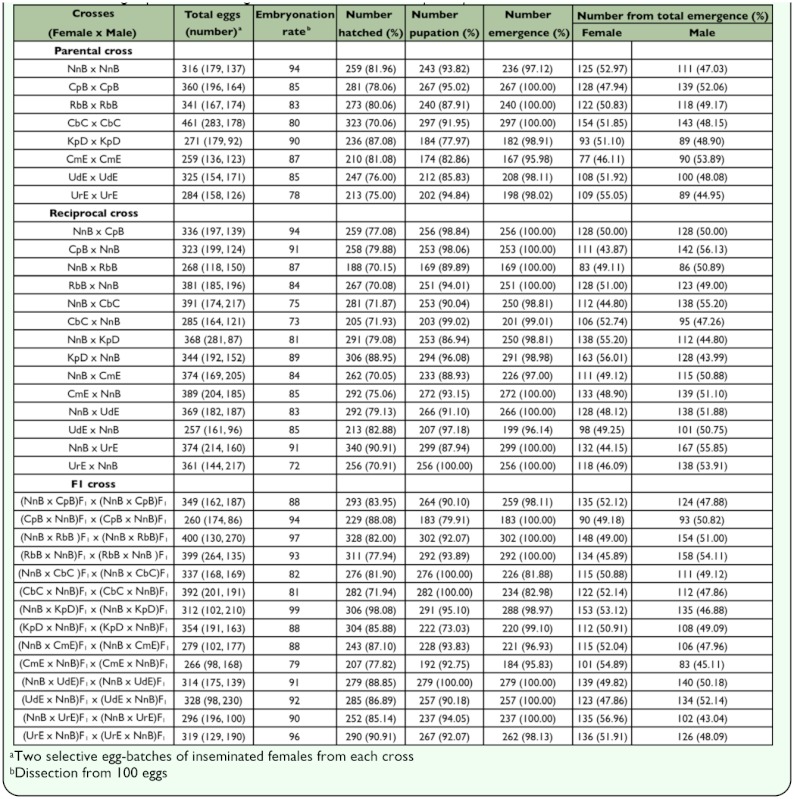
Crossing experiments among the 8 isoline colonies of Anopheles peditaeniatus forms.

Crossing experiments for determining hybrid non-viability, sterility, or breakdown are still a useful tool used in the recognition of anopheline species complexes. Detailed genetic incompatibility, including lack of insemination, embryonation, hatchability, larval survival, pupation, emergence, adult sex distortion, abnormal morphology, and reproductive system are useful criteria for elucidating sibling species or subspecies status (Baimai et al. 1987, 1988; Sawadipanich et al. 1990; Subbarao 1998). However, a point worth noting is that an isoline colony established from the combinative characters of morphological and/or cytological markers has to be seriously considered. A laboratoryraised colony established from a naturally mixed population should be omitted, since it may be a mixture of cryptic species or sibling species. Several intra-taxa of the anopheline species that were primarily detected with cytological differences and/or variations that led to doubt of the status of sibling species or subspecies were subsequently confirmed by crossing experiments. These crossing experiments were for sibling species, e.g. *An. dirus* Peyton and Harrison complex (Baimai et al. 1987, 1988; Sawadipanich et al. 1990), *An. maculatus* Theobald complex (Chabpunnarat 1988, Thongwat et al. 2008), and *An. minimus* complex (Choochote et al. 2002b; Somboon et al. 2005); and subspecies (cytological races), e.g. *An. sinensis* Wiedemann Forms A and B (Park et al. 2008), *An. vagus* Doenitz Forms A and B (Choochote et al. 2002a), *An. pullus* Yamada Forms A and B (Park et al. 2003), *An. aconitus* Doenitz Forms B and C (Junkum et al. 2005), and *An. campestris*-like Form B, E, and F (Thongsahuan et al. 2009).

In this study, 4 tentative karyotypic forms of *An. peditaeniatus*, i.e. Form B (X_2_, X_3_, Y_2_), C (X_3_, Y_3_), D (X_3_, Y_4_), and E (X_2_, X_3_, Y_5_) were obtained from natural populations in Thailand. It is interesting to note that the ancestral Form A (X_2_, Y_1_), reported by Baimai et al. (1993), was not detected in any isoline colonies, as only a few samples appeared to be used in the current study. Even though Form A (X_2_, Y_1_) was not detected in the present investigation markedly distinct characteristics, particularly the Y chromosomes among the 4 karyotypic forms, were enough to perform their genetic proximity thoroughly. Accordingly, the crossing experiments were carried out among the 4 karyotypic forms in order to determine the degree of genetic proximity. In addition, their comparative DNA sequences of ITS2, COI, and COII were included in this study. The results of no post-mating reproductive isolation among the 4 karyotypic forms, by yielding viable progenies and synaptic salivary gland polytene chromosomes through F_2_-generations, suggested their conspecific nature. The very low intraspecific sequence variations (0.0 – 1.1%) of the nucleotide sequences of ribosomal DNA (ITS2) and mitochondrial DNA (COI and COII) of the 4 karyotypic forms were strong supportive evidence. Additionally, the length (463 bp) and sequences of ITS2 regions of *An. peditaeniatus* forms obtained in this study were identical to that of a previous report (Ma and Xu 2005). Similar results have been reported in *An. sinensis* Forms A and B (Park et al. 2008), *An. vagus* Forms A and B (Choochote et al. 2002a), *An. pullus* Forms A and B (Park et al. 2003), *An. aconitus* Forms B and C (Junkum et al. 2005), and *An. campestris*-like Forms B, E, and F (Thongsahuan et al. 2009).
